# Room-Level Fall Detection Based on Ultra-Wideband (UWB) Monostatic Radar and Convolutional Long Short-Term Memory (LSTM)

**DOI:** 10.3390/s20041105

**Published:** 2020-02-18

**Authors:** Liang Ma, Meng Liu, Na Wang, Lu Wang, Yang Yang, Hongjun Wang

**Affiliations:** 1 School of Information Science and Engineering, Shandong University, 266237 Qingdao, China; millilitre@126.com (L.M.); nawang_vc2014@163.com (N.W.); wanglu2018@sdu.edu.cn (L.W.); yyang@sdu.edu.cn (Y.Y.); 2 State Grid Shandong Electric Power Research Institute; Jinan 250002, China; liumengup@126.com

**Keywords:** fall detection, IR-UWB, ConvLSTM, deep learning

## Abstract

Timely calls for help can really make a difference for elders who suffer from falls, particularly in private locations. Considering privacy protection and convenience for the users, in this paper, we approach the problem by using impulse–radio ultra-wideband (IR-UWB) monostatic radar and propose a learning model that combines convolutional layers and convolutional long short term memory (ConvLSTM) to extract robust spatiotemporal features for fall detection. The performance of the proposed scheme was evaluated in terms of accuracy, sensitivity, and specificity. The results show that the proposed method outperforms convolutional neural network (CNN)-based methods. Of the six activities we investigated, the proposed method can achieve a sensitivity of 95% and a specificity of 92.6% at a range of 8 meters. Further tests in a heavily furnished lounge environment showed that the model can detect falls with more than 90% sensitivity, even without re-training effort. The proposed method can detect falls without exposing the identity of the users. Thus, the proposed method is ideal for room-level fall detection in privacy-prioritized scenarios.

## 1. Introduction

With the increasing life expectancy and the declining birth rate, aging populations have become a grave social issue. Providing health and social care for the growing older population is drawing increasing attention. According to a report by the World Health Organization (WHO), millions of fall-induced injuries and deaths occur around the globe each year. Older people have weaker balance, so they have a high risk of falling down [1,2]. As older people are physically frail, so they are vulnerable to the injuries caused by falls [3]. If they cannot get immediate medical assistance, their condition is likely to deteriorate, ultimately resulting in life-endangering illness. Thus, timely calls for help in the event of a fall can make a difference in the well-being of our elders.

When an elder falls and becomes injured, there are two possible scenarios. The first is that the elder is in the company of others or in a public area. In this scenario, the fall event can be easily noticed, and the elder will be likely to get medical assistance in time. The second scenario is that the elder is in a private location and alone. When this happens, the elder must attract the notice of others to achieve help. Clearly, the second scenario is more dangerous for the injured elder. However, in this scenario, given that video-based methods have privacy issues in private locations and the elder may leave any carried devices behind, this problem needs to be approached from a different angle.

The current research on fall detection can be categorized into three groups: wearable-device-based [4], ambient-sensor-based, and camera(vision)-based [5]. Wearable-device-based fall detection methods typically require that the target carries devices like sensors or phones [6,7], while the other two categories of methods do not require the target to carry any extra devices. Device-based methods are less convenient for the users when it comes to the field of fall detection [8]. First, the devices can be left or forgotten, which makes the system fail to function. Since older people tend to have poor memory and even memory loss as their age increases, this is likely to happen. As these devices cause extra burden and inconveniences to people’s lives, people tend to leave those devices behind, especially when they are alone and in private locations where they need help the most in case of a fall. Video-based methods have privacy issues [9] when it comes to deployment in private rooms where, again, fall detection systems are needed most. Thus, a robust device-free fall detection method that does not rely on video surveillance seems to be the best solution for the fall detection problem.

In recent years, radio frequency technology has been applied in areas like localization [10,11,12], activity detection [13,14], and target tracking [15,16]. By capturing the changes of wireless channels that are caused by moving targets, the activities of the target can be monitored without the active participation of the target. Device-free activity recognition methods based on radio frequency can be categorized according to the information they utilize. Research in this area centers around methods that are based on channel state information (CSI), custom equipment, and radar. 

As CSI information can be acquired from many commodity Wi-Fi access points and network interface cards, CSI-based methods take advantage of the widely deployed Wi-Fi infrastructure and detect human activity by measuring the changes of CSI that are caused by the presence of humans. RT-fall [17] uses CSI features and support vector machine (SVM) techniques to detect falls. E-eyes [18] uses CSI histography to recognize walking and staying still. WiFall [19] extracts the statistical features of CSI information, such as standard deviation and signal entropy. However, CSI-based methods have optimal performance when the target is between two Wi-Fi access points, which limits its application value.

Methods based on custom equipment use devices that generate wireless signals that have characteristics that are especially suitable for activity detection, thus providing more fine-grained features of human activities. Wisee [20] used universal software radio peripherals (USRPs) to capture Wi-Fi signal and measure the doppler frequency shift that was caused by the human body to recognize nine different poses. Wision [21] used multipath to construct an image of the surrounding targets. The specially designed hardware that was used in those methods typically has a high cost and is not suitable for large-scale deployment.

Many radar-based methods extract micro doppler features to detect different movement speeds of human body parts. Recently, A. Chelli et al. [22] combined micro doppler features with a cubic SVM and achieved an accuracy of 99.9%. Ultra-wideband (UWB) radar has a high resolution and penetrability due to its wide bandwidth. In the past few years, UWB radar has taken a great leap forward on miniaturization and has greatly lowered its power consumption and cost, making it ideal for applications in smart home environments [23,24]. UWB bistatic radar has been used in research to classify objects that are blocked by foliage with more than 90% accuracy [25,26,27]. In the research of J. Park et al., impulse radio UWB (IR-UWB) was also applied in research on human gesture recognition by combining it with principal components analysis (PCA) and an SVM [28]. Monostatic UWB radars have been combined with neural networks to detect targets and falls [29,30,31], but these studies have focused on sensors that are mounted above doors [29,30] and in restrooms [31], so they can only detect targets and falls in a very limited range. Y. Lin et al. [32] used UWB frequency-modulated continuous wave (FMCW) radar to solve human activity classification problems. Their method had good results but required rather expensive devices.

Most past research on fall detection has utilized handcrafted features and conventional machine learning methods like SVMs. However, since effective fall detection is challenged by the vast variation of observation conditions, environmental factors, and unexpected cases, these methods cannot fulfill the requirements for practical fall detection systems. Recently, deep learning has demonstrated amazing capabilities in areas like computer vision, video processing, and natural language processing. Many researchers have adopted this new tool in fall detection research and acquired good results. The work of Y. Lin et al. [32] used an iterative convolutional neural network (ICNN) followed by random forests to deal with radar signals. Sadreazami et al. employed a deep convolutional neural network [33] and a deep residual neural network [34] to learn features from radar time-series signals. However, those network structures either only dealt with one dimensional radar time-series signals or used CNNs to extract features based on shapes but failed to take advantage of the spatiotemporal structure of the data. In this paper, we approach UWB-radar-based fall detection by combining a CNN and convolutional long short term memory (ConvLSTM) to extract spatiotemporal features from radar ranging data flow. A summary of the recent studies on fall detection systems based on machine learning is shown in Table 1.

In this paper, we propose a whole-room fall detection scheme by using IR-UWB radar that produces one-dimensional data frames. The fall detection problem was transformed into a classification problem of sequences of one-dimensional range data. A network structure combining CNN layers and ConvLSTM is proposed and evaluated in terms of accuracy, sensitivity, and specificity. The experimental results show that our method outperforms CNN-based methods in terms of accuracy and robustness due to its ability to extract both the shape-based features and time-based sequential features.

## 2. Materials and Methods

### 2.1. Data Acquisition with IR-UWB Monostatic Radar

**Figure 1:**
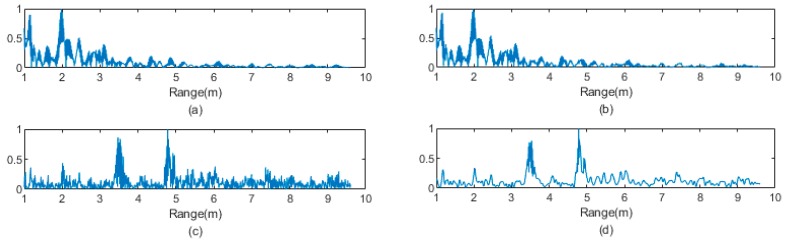
(**a**) Raw echo data that were collected from the impulse–response ultra-wideband (IR-UWB) module and (**b**) reference echo data that were collected when no target was in the monitored area. (**c**) The signal difference that was calculated by subtracting the reference echo data from the raw echo data. (**d**) The result of applying wavelet denoising to the signal difference.

IR-UWB monostatic radar consists of a UWB transmitter and a receiver. For each data frame, the UWB transmitter emits several UWB pulses (p(t)). Pulses bounce back on reflective surfaces in the environment and are sampled by the receiver at a fixed sample rate, $F_{s}$. Signals that have traveled different distances can then be distinguished by their time of arrival (TOA). The received echo signal can be expressed in vector form as

(1) $$R_{t}=\left[R_{t}\left(1\right),R_{t}\left(2\right),\ldots ,R_{t}\left(N\right)\right].$$

where N represents the number of samples in one data frame. A larger N value indicates a longer detection range. A sample of raw received signal by an IR-UWB monostatic radar is shown in Figure 1a. Note that the signal was mapped into [0, 1]. In a typical indoor setting, there are a lot of static reflective surfaces in the environment. These surfaces accounted for the peaks in Figure 1a. Those pikes obscured the signal that was reflected from the target. To solve this problem, a reference datum $R_{Ref}$, taken with no target in the environment, can be subtracted from the raw received signal.

(2) $$\Delta R_{t}=R_{t}-R_{Ref}.$$

As the target enters the detection range of the IR-UWB radar, it introduces more reflective surfaces to the environment. As a result, a strong peak appears in $\Delta R_{t}$ at the location of the target. Due to the effect of multipath in indoor environments, more than one peak can appear, as is shown in Figure 1c. The shape and strength of the peaks contain information on the pose of the target. Taking a step further, the activity of the target can be inferred by extracting features that represent the change of pose in consecutive frames. Then these features can be used to detect falls.

The goal of fall detection by using IR-UWB radar is to use the observed sequential radar echo frames to determine which activity the target is conducting. This problem can be regarded as a spatiotemporal sequence classification problem.

Let *M* be the number of frames each observation contains, $S\subset R^{N\times M}$ be the domain of collected echo data, and L⊂{1,…K}⊂R be the domain of output activity label l (with a total number of K different activities). Then, the fall detection problem can be expressed as

(3) $$\tilde{l_{t}}=argmax\;p(l_{t}|\hat{\Delta R_{t-M+1}},\hat{\Delta R_{t-M+2}},\ldots ,\hat{\Delta R_{t}}).$$

### 2.2. Dataset

**Figure 2:**
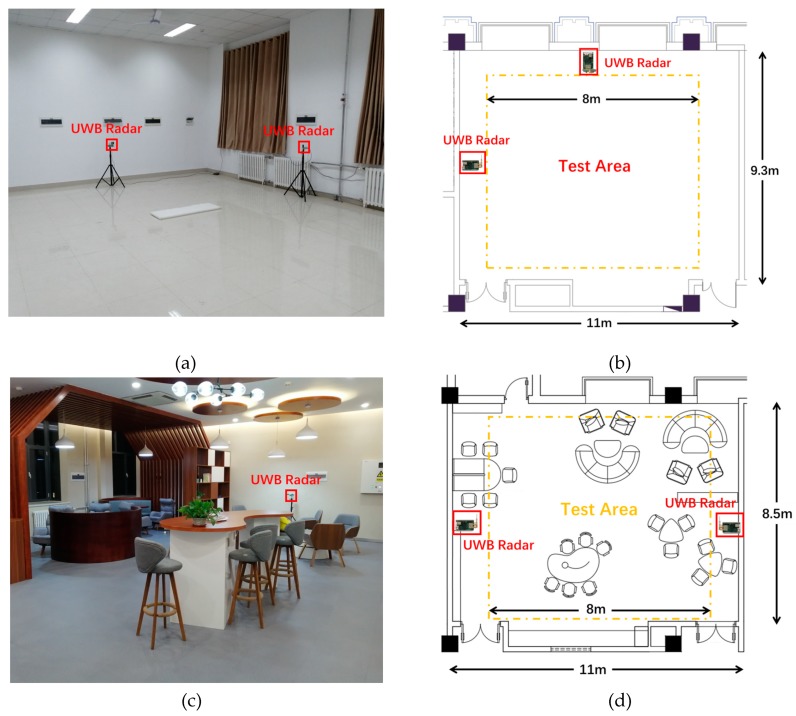
(**a**) A photo of the laboratory environment. (**b**) Floor plan of the laboratory environment. (**c**) A photo of the lounge environment. (**d**) Floor plan of the lounge environment.

**Figure 3:**
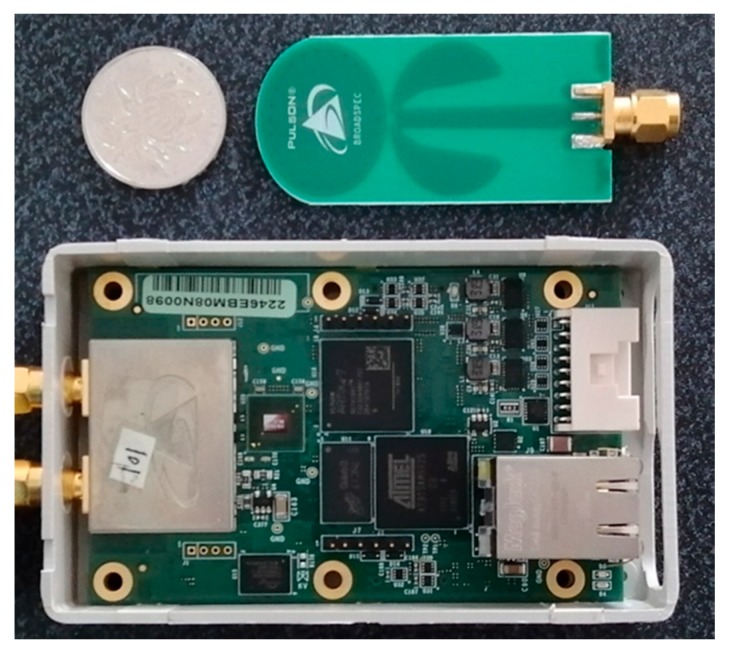
The BroadSpec planar elliptical dipole antenna (top) and Pulson P440 IR-UWB radar (bottom) that we used in our experiments.

In this section, all the data were collected by using an off-the-shelf IR-UWB radar in two indoor environments, including a laboratory and a lounge at the Public Experimental Teaching Center of Shandong University. The environmental settings are shown in Figure 2. The laboratory environment had no furniture, while the lounge environment was heavily furnished. The radar module we used, Pulson P440 [36], transmitted UWB pulses at 30 dBm by using two omnidirectional antennas. Figure 3 shows the IR-UWB radar and the antenna we used. The UWB radar operated at 4 GHz, with a 1.7 GHz bandwidth. The sample rate of the UWB receiver was 61 ps, which enabled a spatial resolution of approximately 0.9 centimeters. The antennas that were used were Time Domain’s BroadSpec planar elliptical dipole antennas with a 3 dBi gain. The UWB modules were mounted on tripods at a height of 1.2 m, which was approximately the height of the torso of the test subjects. In our experiments, two UWB radars were employed to collect data. The UWB radar modules were connected to a PC via network cables. Interference between the radar modules was avoided by letting them take turns transmitting signal and processing data. The frame rate of the raw data flow was 5 frames/s.

**Figure 4:**
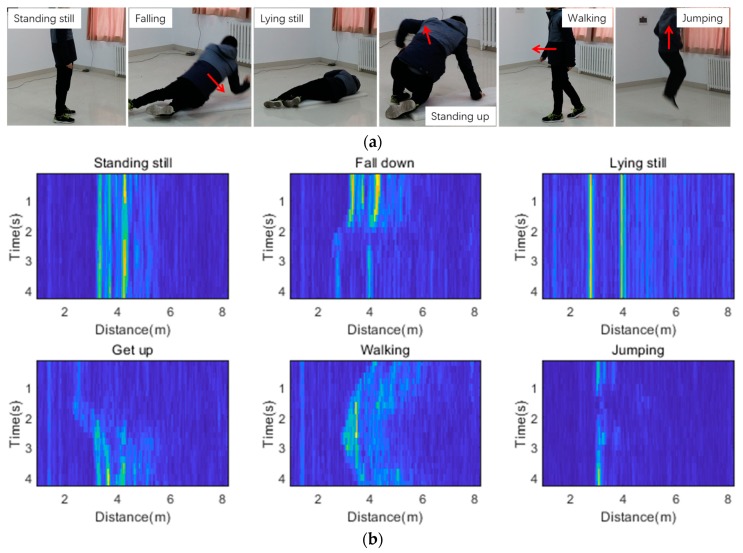
(**a**) The six activities considered in this work. (**b**) Training data examples with 20 frames per sample. Note that the samples in the picture covered the distance from 0.9 to 8.2 m. The size of the samples was 20 × 800.

Five volunteers, including three men and two women, served as test subjects. Basic information of the five subjects are shown in Table 2. To distinguish falls and other daily activities, six different activities were examined including standing still, falling, lying still, standing up, walking, and jumping. Figure 4a shows the six activities we focused on. A reference signal was collected before the targets entered the area of interest. Volunteers conducted the six activities in the test area at random locations and facing random directions. To reduce the possibility of human error, the raw data recordings were manually labelled by three researchers, and the average value of their labelling results was treated as the truth value. The time of recording for each activity sample was 4 s, which meant that each sample contained 20 frames. In the laboratory environment, we collected 102 samples for each one of the five volunteers who performed every activity. Among the 102 samples, 62 (60.7) samples were used to generate the training set, 20 (19.6%) samples were used to form the validation set, and the remaining 20 (19.6%) samples were used to form the test set. A total of 3060 samples were collected. The validation set and the test set contained 600 samples each. Some samples of the dataset are shown in Figure 4b. In the lounge environment, we collected 126 samples for one volunteer who performed every activity. In this environment, a total number of 756 samples were collected. All the data that were collected in the lounge were used to form test set 2. The dataset is available online [37].

### 2.3. Preprocessing

As can be seen in Figure 1c, the signal difference was noisy. First, the raw echo data that were collected by the UWB monostatic radar were normalized into [0, 1]. To eliminate the effect of noise, a wavelet filter was used to denoise each scan data. We used Daubechies5 wavelet, as it is a compactly supported wavelet with an extremal phase and the highest number of vanishing moments for a given support width [38]. It is ideal for smoothing a noise-cluttered signal. An example result of the denoising process is shown in Figure 1d.

In our experiments, we found that, sometimes, very high peaks existed in $\Delta R_{t}$. After normalization, these peaks obscured all other features in the result, which was detrimental for extracting robust features of a target’s activities. As a result, very little useful information could be learned during the training phase. Thus, after denoising, the amplitude of the result was transformed into logarithmic coordinate before being mapped into [0, 1]. In this way, features with low amplitude were enhanced and the robustness of the system was improved.

**Figure 5:**
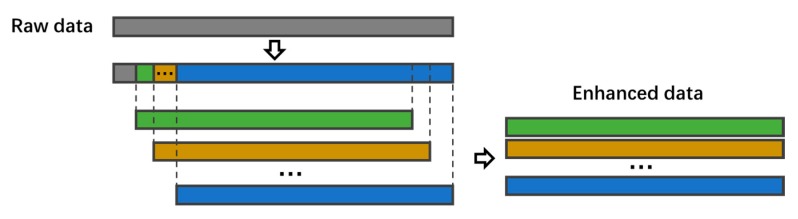
The training data were augmented by moving the sample window along the distance axis with a fixed step length.

The training data were then augmented by linear translation of the sampling window. An illustration of the linear translation we employed is shown in Figure 5. The sampling window moved in the direction of the range axis with a predetermined step, and multiple training samples were generated with one raw measured data sample. After augmentation, the training set contained 9300 samples.

### 2.4. Convolutional LSTM

**Figure 6:**
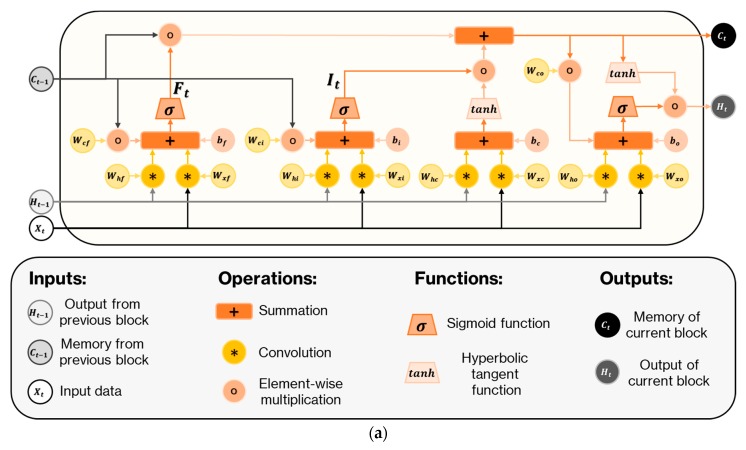

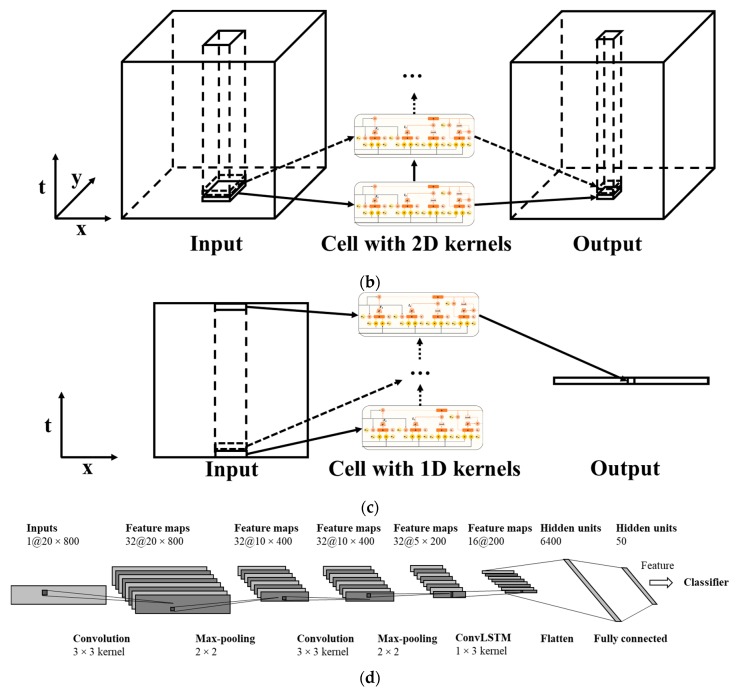
(**a**) Example of a convolutional long short time memory (ConvLSTM) cell. (**b**) Structure of the ConvLSTM that was proposed in [39]. (**c**) Structure of the ConvLSTM that was used in our method. (**d**) Structure of the proposed network. The size of the input data was 20 × 800.

As a special Recurrent neural network structure, LSTM is a powerful tool for modeling long sequences. The core contribution of an LSTM structure is the introduction of memory cells that accumulate or discard state information through dynamic gates. LSTM has been used in various studies to model long-range correlations. However, spatiotemporal data has to be converted into 1D sequences when serving as input of the LSTM structure, thus losing some of their structural information. To solve this problem, Xingjian Shi et al. proposed the ConvLSTM structure [39]. By introducing convolution into the classic LSTM structure, their solution can extract spatial information as well as temporal information. In contrast to the classic LSTM structure, ConvLSTM updates the state of a certain cell by the current and past states of a small reception field. The update process of ConvLSTM can be expressed as

(4) $$I_{t}=\sigma \left(W_{xi}*X_{t}+W_{hi}*H_{t-1}+W_{ci}\circ C_{t-1}+b_{i}\right).$$

(5) $$F_{t}=\sigma \left(W_{xf}*X_{t}+W_{hf}*H_{t-1}+W_{cf}\circ C_{t-1}+b_{f}\right)$$

(6) $$C_{t}=F_{t}\circ C_{t-1}+I_{t}\circ tanh\left(W_{xc}*X_{t}+W_{hc}*H_{t-1}+b_{c}\right)$$

(7) $$O_{t}=\sigma \left(W_{xo}*X_{t}+W_{ho}*H_{t-1}+W_{co}\circ C_{t}+b_{0}\right)$$

(8) $$H_{t}=O_{t}\circ \tan h\left(C_{t}\right)$$

where $X_{t}$ is the input, $C_{t}$ is the cell output, $H_{t}$ is the hidden state, $I_{t},\;F_{t},O_{t}$ are the gates, $W_{x},\;W_{t},W_{c}$ are convolutional kernels, $b_{i},\;b_{f},b_{c}$ are the biases of the gates, ‘∗’ denotes the convolution operator, and ‘∘’ denotes element-wise multiplication. The structure of the ConvLSTM cell is illustrated in Figure 6a. Note that in the ConvLSTM structure, the inputs, outputs, states, and gates are all 3D tensors instead of vectors, as in a standard LSTM structure. In the input-to-state and state-to-state transitions, product operations are replaced with convolution operations. The ConvLSTM structure was initially proposed to process video data in which each frame is a 2D image, so it employed 2D convolutional operations to enhance the standard LSTM structure such that it gained a small reception field for the two spatial dimensions. In our case, however, each frame had only one spatial dimension. Thus, 1D kernels were used in the convolutional LSTM layer.

Before training, all the weights were randomly and orthogonally initialized. The Nesterov adaptive momentum (Nadam) optimizer [40] was utilized to train the model. When training and testing, data were segmented into mini-batches of 100 data samples. The accumulated gradient of each batch was computed and used to update the parameters. Dropout layers and $L^{2}$ regularizers were utilized to prevent overfitting.

The network we propose had two 2D convolution layers—a 1D convolutional LSTM layer followed by a dense layer. In this network, the 2D convolution layers are used in the same way that 3D convolution layers are used in solving video classification problem. The convolution layer uses 3 × 3 kernels, followed by max-pooling layers with a scaling of 2. The convolution layers with a small reception field followed with pooling layers can extract low-level spatiotemporal features and reduce the number of parameters that need to be trained in the following layers. The output of the CNN layer is then fed into a 1D convolutional LSTM layer. Rather than returning the full sequence, the ConvLSTM layer is set to the mode where only the last output in the sequence is returned. Figure 6b,c illustrates the differences between the ConvLSTM structure proposed in [39] and the one that we employed in our method. The dense layer immediately after the ConvLSTM layer has 64 hidden neurons. The proposed network structure is shown in Figure 6d. The total number of trainable parameters of the proposed network structure is 172,836. The total number of trainable parameters of Lenet-5 network with the same input is 1,027,026.

First, we employed two CNN layers to automatically define and extract features. Then, by adding a convolutional LSTM layer, the temporal structure of the falling process could be learned by the network.

## 3. Results and Discussion

The effectiveness of the proposed fall-detection method is shown in terms of accuracy, sensitivity, and specificity [41]. The metrics can be expressed as

(9) $$accuracy=\frac{TP+TN}{TP+FP+TN+FN}.$$

(10) $$sensitivity=\frac{TP}{TP+FN}.$$

(11) $$specificity=\frac{TN}{TN+FP}.$$

where TP is the number of fall samples identified as falls, FP is the number of non-fall samples identified as falls, TN is the number of correctly classified non-fall samples, and FN is the number of falsely classified non-fall samples. In our experiments, all the activities other than falling were considered non-falls. Non-fall samples must be classified as the corresponding activity to contribute to TN count. We compared the proposed method with the classic Lenet-5 architecture, which has been adopted in similar research [32,35]. Three kinds of classifiers, SoftMax, K-nearest neighbors (KNN), and random forest (RF), were selected to test the performance of the proposed method when using different classifiers. In the training process, the drop rate of dropout layers was set to 0.5, and the penalty of the $L^{2}$ regularizers was 0.1. The learning rate was set to 0.001, and the number of maximum epochs was set to 1000 with an early stopper enabled.

### 3.1. Performance Evaluation

The performance comparison of methods is shown in Table 3. The data from the lab environment were used in this evaluation. By combining the CNN and ConvLSTM, the proposed method achieved a better overall performance than the CNN-based network structure. Among the classifiers, the SoftMax classifier had the best performance in terms of accuracy and specificity. Please note that the classification task was challenging because that the radar had a low frame rate and the test subjects performed the activities in randomized locations and with different facings. Despite the challenges, the proposed method was still able to show good performance, and it was comparable to state-of-the art methods.

**Figure 7:**
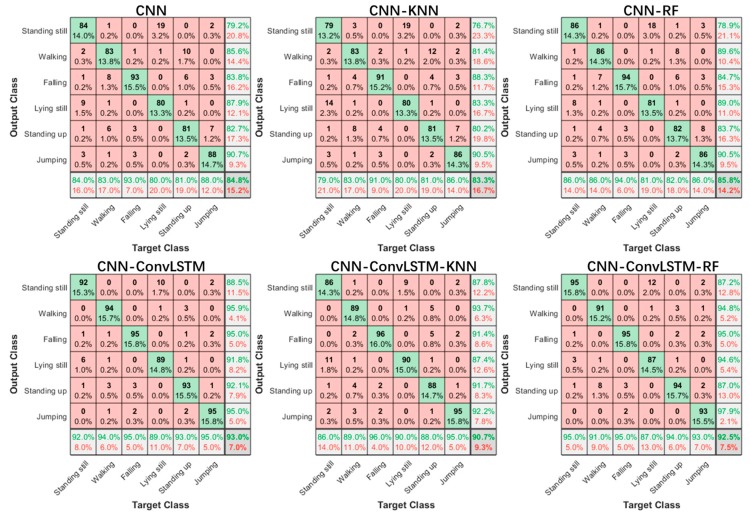
Confusion matrices of six activities.

Figure 7 shows the confusion matrices of the classification result of six activities when using different methods. Generally, our methods performed better than the CNN-based methods. It is interesting to note that standing still and lying still were the easiest to confuse. This might have been due to the lack of robust features to distinguish between the two activities, as the most appreciable differences between the two activities were the power of the reflected signal, rather than spatiotemporal motions. Compared with the CNN-based approach, the ConvLSTM-based approach has an advantage in identifying movement-based activities. This is because ConvLSTM layers have the ability to correlate long time sequences while the convolution operation makes it possible to gain a larger area of perception than the classic LSTM structure. 

### 3.2. Effect of the Number of Frames per Sample

**Figure 8:**
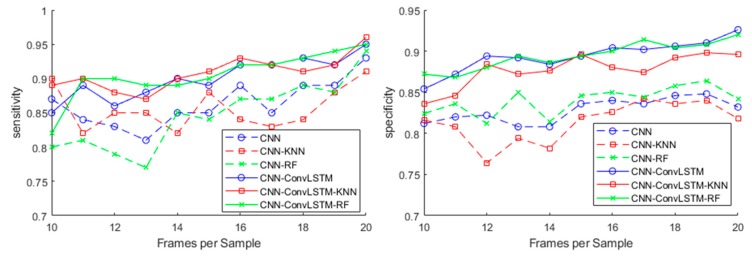
The effect of frames per sample on the sensitivity and specificity of the methods.

The effect of the number of frames per sample on the sensitivity and specificity of the methods is shown in Figure 8. The data from lab environment were used in this test. The datasets with fewer frames were generated from the dataset with the maximum number of frames by alternatively removing frames from the two ends of each sample. It can be concluded from the figure that our method constantly yielded better results than the CNN-based methods. The best result was achieved when each sample contained 20 frames. 

Intuitively, as the number of frames per sample increased, the samples contained more transition information about the different poses that the target person assumed before and after the activity. The transition information could be utilized by learning algorithms to gain better results. This was generally true when each sample contained no more than 16 frames. However, the performance of the investigated methods may become worse beyond that point. This may be caused by the introduction of redundant information that reduces the robustness of the extracted features. Note that the frame rate of the system was about 5 frames/s. Thus, 20 frames covered a time span of 4 s. In our experiments, falls generally took 1–2 s, while the activity of standing up took 2–4 s.

### 3.3. Evaluation of Transferability to Other Environment

**Figure 9:**
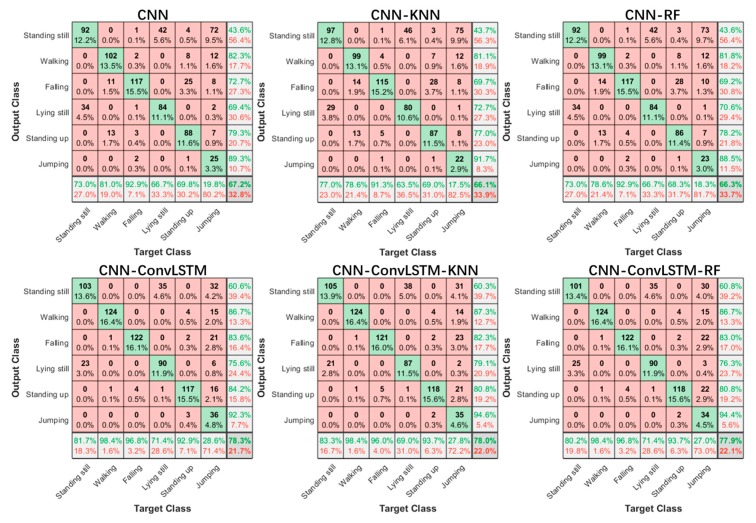
Confusion matrices of six activities where the model was used to classify the samples collected in the heavily furnished lounge.

Table 4 shows the accuracy, sensitivity, and specificity of the trained model when predicting the data that were collected in the heavily furnished lounge environment. The models were trained with the data from the lab environment and tested with the data from the lounge environment. The confusion matrix of the six activities is shown in Figure 9. The model trained in Section 3.1 and the data from the lounge environment were used in this evaluation. It can be concluded that the proposed method could still detect falls with a high sensitivity even when the environment was changed. However, the trained model showed a poor performance when classifying the jumping samples in the new environment. This was because with the furniture in the environment, the movements of some body parts of the target person may have gotten blocked, especially the movements of the person’s legs. As the model heavily relied on the activities of the legs to classify jumping activity, its performance was heavily influenced. 

### 3.4. Evaluation of Performance Classifying Activities of Unknown Subjects

The data set from all five people were trained by five-fold cross-validation in a leave-one-subject-out fashion, and the result is shown in Table 5. Specifically, each time a model was trained, the data of four test subjects were used for training and validation and the data of the remaining person were used for testing. The data samples that belonged to one test subject whose data were used for training and validation were divided into four folds—three folds for training and the remaining fold for validation. The result showed that the performance of the proposed method remained stable when classifying activities of previously unknown subjects.

Table 6 shows the accuracy of the schemes in classifying the five subjects by using deep learning methods. The dataset that was used in this test was the same as that in Section 3.1, but the label was changed to the serial number of the corresponding test subject. It seems that the proposed method was sensitive to different activities but was insensitive to differences of individuals. This is good for preserving the privacy of the users, because it can provide the fall detection service with a low risk of exposing the identity of the users.

### 3.5. Evaluation of Execution Time

Table 7 shows the execution times of the models. Note that this result was achieved on a laptop with an Intel i5-7300 central processing unit (CPU) and a 2.6 GHz main frequency. The values were calculated as the average execution time for 100 classification tasks. Though the proposed method had a longer execution time compared to the CNN-based models, since the frame rate of the UWB radar we used was 5 Hz, the laptop could provide real-time service for the classification tasks with less than 0.5% CPU time.

## 4. Conclusions

In this paper, a room-level fall detection scheme based on IR-UWB monostatic radar was proposed. After preprocessing and the data augmentation of the raw data, the fall detection problem was transformed into a spatiotemporal sequence classification problem. To solve this problem, we devised a deep learning scheme based on the combination of a CNN and 1D ConvLSTM layers. We evaluated the performance of our method against the CNN-based methods that have been used in similar studies. In terms of accuracy, sensitivity, and specificity, the experimental results suggest that the proposed method had a better and more robust performance than the CNN-based methods. Further tests in a lounge environment showed that the model could still detect falls with high sensitivity in heavily furnished environments. Experiments showed that the proposed method could provide accurate real-time fall detection service with a low risk of exposing the identity of the users. The proposed method is ideal for deployment in private rooms.

In future work, the influence of the environment should be further investigated to enhance the robustness of the extracted features.

## Figures and Tables

**Table 1:** Summary of recent studies about fall detection systems based on machine learning techniques.

Ref	Type of Sensor	N Users	N Records	N Classes	Covered Area	Algorithms	Accuracy (%)	Sensitivity (%)	Specificity (%)
[32]	FMCW Radar	5	6720	7	6 m	ICNN Random Forest	99.11	97.78	99.45
[30]	UWB radar	2	240	6	Door area	SEP SVM	97.5	100	NS
[19]	Wi-Fi	10	13,000	4	7 m	SVM Random Forest	94	NS	NS
[35]	Surface electromyography	10	1600	4	NS	IDPC-CNN	92.55	95.71	91.7
[34]	UWB radar	10	336	5	10 m	Deep residual neural network	93.07	90.91	NS
[22]	Simulated Wi-Fi using path model	30	900	3	NS	Cubic SVM	98.7	99.7	NS
[33]	UWB radar	5	2665	5	10 m	CNN	95.3	100	91.67

**Table 2:** The gender, weight, and height of the test subjects.

Test Subjects	I	II	III	IV	V
Gender	Male	Male	Female	Male	Female
Weight	60 kg	90 kg	50 kg	75 kg	55 kg
Height	175 cm	180 cm	160 cm	180 cm	165 cm

**Table 3:** Accuracy, sensitivity, precision, and specificity of the fall detection schemes.

Method	CNN	CNN-KNN ^1^	CNN-RF	CNN-ConvLSTM	CNN-ConvLSTM-KNN ^1^	CNN-ConvLSTM-RF
Accuracy	84.8%	83.3%	85.8%	**93.0%**	90.7%	92.5%
Sensitivity	93.0%	91.0%	94.0%	95.0%	**96.0%**	95.0%
Specificity	83.2%	81.8%	84.2%	**92.6%**	89.6%	92.0%

^1^ K = 3 for all the KNN classifiers mentioned in the paper.

**Table 4:** The accuracy, sensitivity, precision, and specificity of the trained model that classified the samples that were collected in the heavily furnished lounge.

Method	CNN	CNN-KNN	CNN-RF	CNN-ConvLSTM	CNN-ConvLSTM-KNN	CNN-ConvLSTM-RF
Accuracy	67.2%	66.1%	66.3%	78.3%	78.0%	77.9%
Sensitivity	92.9%	91.3%	92.9%	96.8%	96.0%	96.8%
Specificity	62.1%	61.1%	61.0%	74.6%	74.4%	74.1%

**Table 5:** Accuracy, sensitivity and specificity of the schemes in the leave-one-subject-out cross validation.

Method	CNN	CNN-KNN	CNN-RF	CNN-ConvLSTM	CNN-ConvLSTM-KNN	CNN-ConvLSTM-RF
Accuracy	84.24	83.01	86.41	**95.78**	93.33	95.36
Sensitivity	93.73	91.76	91.96	**98.04**	97.65	97.06
Specificity	82.35	81.25	85.29	**95.33**	92.47	95.02

**Table 6:** Accuracy of the schemes when classifying the five subjects.

Method	CNN	CNN-KNN	CNN-RF	CNN-ConvLSTM	CNN-ConvLSTM-KNN	CNN-ConvLSTM-RF
Accuracy	17.9	19.6	18.6	19.3	22	21.7

**Table 7:** Comparison of the execution times of the models.

Method	CNN	CNN-KNN	CNN-RF	CNN-ConvLSTM	CNN-ConvLSTM-KNN	CNN-ConvLSTM-RF
Execution time/task (μs)	352	518	367	651	925	662

## Abbreviations

IR-UWB	Impulse–Response Ultra-Wideband
ConvLSTM	Convolutional Long Short Term Memory
CNN	Convolutional Neural Network
WHO	World Health Organization
CSI	Channel State Information
SVM	Support Vector Machine
USRP	Universal Software Radio Peripheral
PCA	Principal Components Analysis
FMCW	Frequency-Modulated Continuous Wave
ICNN	Iterative Convolutional Neural Network
SEP	SEPeration
NS	Not Specified
IDPC-CNN	Improved Dual Parallel Channel Convolutional Neural Network
TOA	Time of Arrival
KNN	K-Nearest Neighbors
RF	Random Forest
CPU	Central Processing Unit
